# 

**Published:** 1954

**Authors:** 


### THE MEDICAL JOURNAL OF THE SOUTH-WEST

*Incorporating*

*The Bristol Medico-Chirurgical Journal*

ARMED FORCES

MAY 24 1954

MEDICAL LIBRARY

1954

